# Supplementary material for: Search for Novel Lead Inhibitors of Yeast Cytochrome bc1, from Drugbank and COCONUT
Source: Molecules. 2021 Jul 16;26(14):4323. doi: 10.3390/molecules26144323 (PMC8307329; doi:10.3390/molecules26144323)
Supplement: Supplementary file 1 [file molecules-26-04323-s001.zip › molecules-1275410 pdf files/Supplementary_files/Supplementary_file_from_Gold_to_MD_and_orca.pdf]

1. Save Gold complex with highest HSC conformation or top SHB conformation according to the manuscript into the pdb file.

2. Open the GaussView and delete ligand and two heme groups from the complex.

3. Open 'R' program and write EXACTLY the following:

```
library(bio3d)

t<-read.pdb('B:/Ozren/COCONUT/Gold_izabr/Gold_izabr_fileovi/To_MD_simul/prot/Only_protein_gold_soln_CNP0406621_27_HBGlu272.pdb') #route to the only_protein_file

FE<-as.matrix(t$atom)

FEsort<-FE[order(FE[,7]),]

FEsort2<-FEsort[,-16]

FEsort2<-FEsort2[,-8]

FEsort2<-FEsort2[,-4]

FE3<-FEsort2

for (i in 1:nrow(FE3)) {

if (FE3[i,1]=='HETATM')

FE3[i,1]<-'ATOM'

FE3[i,2]<-i

FE3[i,10]<-1.00

FE3[i,11]<-0.00

FE3[i,12]<-'CP1'

}


write.table(FE3,
'B:/Ozren/COCONUT/Gold_izabr/Gold_izabr_fileovi/To_MD_simul/prot/output_166.pdb'
,quote=FALSE,sep = "  ",col.names=FALSE,row.names=FALSE)
```

q()

n

4. Open the python and write exactly the following:

```
python

s=''

with open('output_166.pdb','r') as f:

  for line in f:

    data=line.split()

    s+= '{0[0]:<5}{0[1]:>6}'.format(data)+'
'+'{0[2]:<5}{0[3]:<4}{0[4]:<2}{0[5]:>3}{0[6]:>12}{0[7]:>8}{0[8]:>8}{0[9]:>6}{0[10]:>6}
{0[11]:>9}{0[12]:>3}'.format(data)+'\n'



f6=open('w166.pdb','w')

f6.write(s)

f6.close()

exit()
```

5. Add at the beginning of the file ''REMARK original generated coordinate pdb file'' and add at the end of the file: "END". Rename the file according to the docked ligand name.

6. Open the finally obtained file with the GaussView to check it.
