# Supplementary material for: Search for Novel Lead Inhibitors of Yeast Cytochrome bc1, from Drugbank and COCONUT
Source: Molecules. 2021 Jul 16;26(14):4323. doi: 10.3390/molecules26144323 (PMC8307329; doi:10.3390/molecules26144323)
Supplement: Supplementary file 1 [file molecules-26-04323-s001.zip › molecules-1275410 pdf files/Supplementary_files/Supplementary_information_list_1.pdf]

List of 23 pre-step 5 hit compounds (which successfully completed MD simulations), marked (with *) are those which are lead compounds (successfully completed QM/MM) :

DB07831*

DB07943

DB08439*

DB08557*

DB07181

DB14668*

DB12812[a]

CNP0048157

CNP0049103

CNP0061106*

CNP0083976*

CNP0105092

CNP0151817

CNP0153569*

CNP0167760

CNP0191444

CNP0295631

CNP0304461*

CNP0330896

CNP0361420*

CNP0364174

CNP0364473*

CNP0375239

[a] See Supplementary information note 1 regarding this compound.
