# Supplementary material for: Search for Novel Lead Inhibitors of Yeast Cytochrome bc1, from Drugbank and COCONUT
Source: Molecules. 2021 Jul 16;26(14):4323. doi: 10.3390/molecules26144323 (PMC8307329; doi:10.3390/molecules26144323)
Supplement: Supplementary file 1 [file molecules-26-04323-s001.zip › molecules-1275410 pdf files/Supplementary_files/Supplementary_information_note.pdf]

Only for the Drugbank data set, we laso conducted the alternative filtering selection concerning descriptor similarity filtering, which considered the case if all top three energy conformations of the Drugbank have similarity of at least 0.80 (as 80% of 280 descriptors) but under some additional conditions. Besides that at least one X-C=O group is required, the compound may not have any charged functional group at pH 7.4 (as this is also the case for fungicides), and a higher Gold Chemscore fitness cut-off is required for such compound to be considered as a hit compound.

Among 763 Drugbank compounds that had passed fingerprint similarity condition, 37 have passed alternative pipeline (but before Gold docking). When these compounds were submitted to flexible docking, only two DB compounds have passed from the alternative pipeline to MD simulations with HSC higher than the third top-scored fungicide (fenaminstrobin, Chemscore 41.2), and with SHB conformation (DB12812 (Chemscore 48.95) and BD07174 (Chemscore 47.17)), the rest from the alternative pipeline (35 out of 37) are discarded from further consideration.

Table N1. Gold Chemscore Fitness table of the highest scored conformation (HSC) and of the best scored specific H-bond (SHB) (ligand C=O...H–N Glu-272) conformation for alternative filtering selection

Compound	HSC	SHB
DB07274(d)	47.2	42.9
DB12812(d)	49.0	49.0t

(d) For hit compounds obtained by alternative pipeline (which is 3×0.80 similarity instead of 0.95)

Table N2. Molecular dynamics result H-bond table. By default shown are compounds with SHB conformations (unless stated that it is about HSC*). 'Total H2.2' represent percentage of time H-bond (X…H–X < 3.2 Å, X…H < 2.2 Å) is established in MD trajectory, 'H2.2 Glu272' and 'H3.0 Glu272' are respectively defined as strong and weak H-bonds (X…H–X < 3.2 Å, X…H < 2.2 Å and X…H–X < 4,0 Å, X…H < 3.0 Å) between ligand atoms (O,N,Cl,F) and H-N of Glu-272.

Compound	Total H2.2	H2.2 Glu272	H3.0 Glu272
DB07274	6.27	0.07	1.55
DB12812	82.24	0.27	1.78

Table N3. Average interaction energy between ligand and protein through the whole MD (1 ns) trajectory. By default shown are compounds with SHB conformations (unless stated that it is about HSC[(a)] or rigidly docked conformation[(b)]) Protein-ligand interaction energy (E(int)) is calculated according to Ref. [16] (HAC denotes heavy atom count).

Compound	HAC	E(int)/kJ/mol	E(int)/HAC
DB07274	31	-192±13	-6.19±0.42
DB12812	30	-204±17	-6.80±0.57

From tables N2 and N3, DB07274 has all H-bond statistics below 10% interaction frequency and abs(E(int)/HAC) < 6.6, and is therefore discarded from further consideration. On the other hand DB12812 has Total H2.2 > 10% and abs(E(int)/HAC) > 6.6 and can be included into QM/MM simulations.

Table N4. QM/MM binding energy table. ΔEbind as electronic binding energy, Escore is the same scaled by heavy atom count (HAC), ΔG is Gibbs-free binding energy with all enthalpy and entropy corrections to ΔE bind

	B97-D3/def2-SVP//B97-D3/ def2-SVP				B97-D3/def2-SVP//B97-D3/3-21G			
Compound	HAC	ΔE bind	Escore ΔE/HAC	ΔG	HAC	ΔE bind	Escore ΔE/HAC	ΔG
DB12812	30	-27.0	-0.90	+2.5	30	-22.8	-0.76	+15.3
Average for QoI	-	-41.1	-1.45	-25.5	-	-36.1	-1.27	-13.7
Error margin[(a)]	-	6.0	0.2	>6	-	5.5	0.18	>6

Due to the lower score than average for QoI (minus error margin) DB12812 has not passed the last QM/MM step. But it will be mentioned in supplementary information list as the compound which passed first four in-silico steps.
