# Supplementary material for: Search for Novel Lead Inhibitors of Yeast Cytochrome bc1, from Drugbank and COCONUT
Source: Molecules. 2021 Jul 16;26(14):4323. doi: 10.3390/molecules26144323 (PMC8307329; doi:10.3390/molecules26144323)
Supplement: Supplementary file 1 [file molecules-26-04323-s001.zip › molecules-1275410 pdf files/Supplementary_files/Supplementary_Table_S3_rev.pdf]

Table S3. Number of conformations (out of 100 possible) with H-bond interaction, X…H–N < 3.2 Å, X…H < 2.2 Å, X is ligand's H-bond acceptor atom only O, N, F or Cl with backbone amide nitrogen of Glu-272 (the number in parenthesis is the number of cases where ligand's acceptor atom is exclusively oxygen atom)

Compound	Number of conf.	Compound	Number of conf.	Compound	Number of conf.
azoxystrobin	14 (5)	picoxystrobin	2* (1)	famoxadone	1 (1)
metominostrobin	4 (4)	pyrametostrobin	6 (6)	coumoxystrobin	1 (1)
pyraclostrobin	8 (8)	pyribencarb	12 (12)	enoxastrobin	2 (2)
fenamidone	0	triclopyricarb	9 (9)	mandestrobin	16 (16)
kresoxim-methyl	3 (3)	fluoxastrobin	1 (1**)	dimoxystrobin	6 (6)
trifloxystrobin	6 (5)	pyraoxystrobin	2 (2)	flufenoxystrobin	1** (0)
orysastrobin	8 (7)	fenaminstrobin	2** (2)	metyltetraprole	41 (38)
Hits:					
DB03788	0	DB07809	1 (0)	DB08439	11 (11)
DB04930	1* (0)	DB07831	3 (3)	DB08557	7 (7)
DB07181	9 (9)	DB07943	2 (2)	DB08639	0
DB07227	3 (0)	DB08121	0	DB09199	0
DB07244	0	DB08242	4 (4)	DB08384	2
DB14668	10 (10)				

* only under loose H-bond interaction definition of X…H–N < 3.6 Å, X…H < 2.6 Å.

** only under very loose H-bond interaction definition of X…H–N < 4.0 Å, X…H < 3.0 Å. For flufenoxystrobin only Cl atom formed H-bond with amide Glu-272, so docking was repeated with 200 ga runs (and by selecting all atoms within 20 Å instead of 30 Å) and the result was much better including top conformation HSC having carbonyl H-bond with amide Glu-272
