# Supplementary material for: Search for Novel Lead Inhibitors of Yeast Cytochrome bc1, from Drugbank and COCONUT
Source: Molecules. 2021 Jul 16;26(14):4323. doi: 10.3390/molecules26144323 (PMC8307329; doi:10.3390/molecules26144323)
Supplement: Supplementary file 1 [file molecules-26-04323-s001.zip › molecules-1275410 pdf files/Supplementary_files/Supplementary_Table_S8_rev.pdf]

Table S8. Molecular dynamics frequency of interactions table per snapshot for 10000 trajectory points (this means that 10000 in table equals one interaction per snapshot). Contacts here are defined as between any ligand atoms and protein atoms, distance within < 3.5 Å (except Ar-O* Tyr132 where only ligands' O, N, Cl, F and H atoms were considered). The last column represents how much has the ligand's position varied relative to the backbone protein

Compound	CD-Ile125	ArC1-Phe129	ArC4-Phe129	Ar-O* Tyr132	CA-Gly143	ArC1-Phe-278	ArC4-Phe278	CE-Met295	RMSD/Å to backbone
azoxystrobin*	205	4089	3453	2629	10281	6122	152	3810	2.27
metominostrobin	9	4148	25	11365	8843	2	0	4159	1.56
Pyraclostrobin	493	760	6381	1422	14900	2911	116	7713	1.82
kresoxim-methyl	0	94	241	14137	21075	0	0	4130	2.60
trifloxystrobin	13	4951	8657	2818	11923	1677	1	3296	2.81
orysastrobin	629	8990	14735	6595	13625	3	0	12092	2.49
picoxystrobin	527	7547	1134	0	17577	29	0	5243	2.03
pyrametostrobin	3624	8264	382	4106	14935	10668	2846	5571	1.63
pyribencarb	825	3484	3053	0	7617	1740	49	6050	2.22
triclopyricarb	3497	11223	776	870	1212	11493	3273	6026	2.16
Stigmatellin	2222	12613	1675	13761	11062	2436	94	19512	2.98
methyltetraprole	331	988	1651	3647	9624	596	4	3448	1.71
famoxadone	2643	1475	1	6351	12949	0	0	5468	2.16
pyraoxystrobin	10	2906	4	1848	17156	0	0	9923	2.78
fenaminstrobin	1971	10737	15974	5175	16078	6874	116	9744	1.42
coumoxystrobin	126	13786	916	6242	9570	0	0	10145	2.55
enoxastrobin	762	502	3656	171	17387	3163	120	3414	2.31
mandestrobin	39	17475	17517	1825	16948	1	0	11225	2.20
dimoxystrobin	0	15688	1077	1823	11595	3	0	9717	2.32
flufenoxystrobin**	13	6248	711	13021	9972	7	0	4377	2.05
Lead compounds									
DB07831[(a)]	7005	15144	6508	2545	17831	9521	512	11647	2.4
DB08439 (C=O)	8	6724	5754	210	4839	567	0	2767	1.42
DB08557	337	15623	665	0	13882	906	25	4809	1.92
DB14668	13041	495	19631	906	5233	16823	320	7202	2.38
CNP0061106	599	59	1	3474	16603	6985	456	7566	1.48
CNP0083976	292	4159	6	9382	4219	0	0	3638	1.95
CNP0105092	117	7975	2351	600	12077	8	0	8159	2.16
CNP0153569	42	3420	16415	9023	4723	780	0	4180	2.93
CNP0330896	0	66	6168	544	11981	18	0	7125	1.71
CNP0361420	74	2524	18026	572	11218	620	2	14563	2.60
CNP0364473	394	2428	595	5945	10504	286	0	8113	1.83
