# Supplementary figures and images for: Search for Novel Lead Inhibitors of Yeast Cytochrome bc1, from Drugbank and COCONUT
Source: Molecules. 2021 Jul 16;26(14):4323. doi: 10.3390/molecules26144323 (PMC8307329; doi:10.3390/molecules26144323)

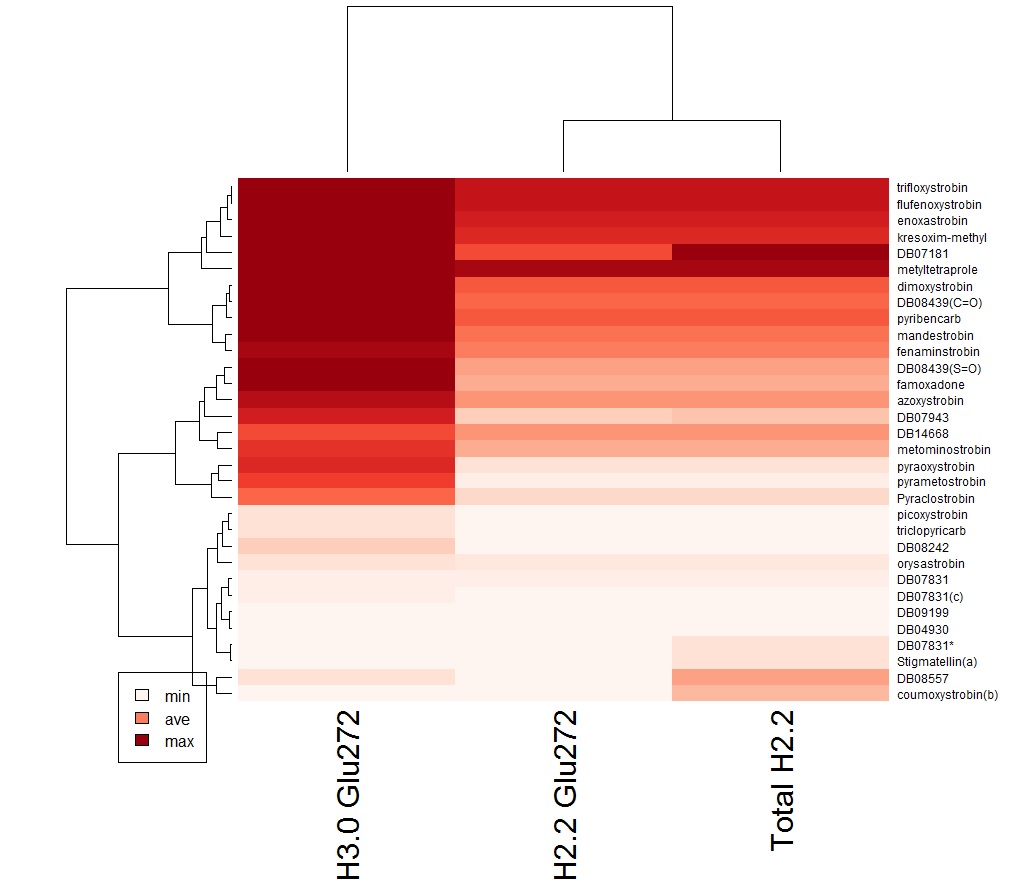

Supplement: Supplementary file 1 [file molecules-26-04323-s001.zip › molecules-1275410 pdf files/Supplementary_files/Supplementary_figure_1.jpg]
