# Supplementary material for: Search for Novel Lead Inhibitors of Yeast Cytochrome bc1, from Drugbank and COCONUT
Source: Molecules. 2021 Jul 16;26(14):4323. doi: 10.3390/molecules26144323 (PMC8307329; doi:10.3390/molecules26144323)
Supplement: Supplementary file 84 [file molecules-26-04323-s001.zip › molecules-1275410 pdf files/Supplementary_files/Supplementary_MD_instructions/Amber_mmpbsa_in/tleap_instructions.odt]

ambpdb -p 0_80_10_pH7.4_QMMM_kresoxim-methyl_HB272_babel.top -c 0_80_10_pH7.4_QMMM_kresoxim-methyl_HB272_babel.crd > 0_80_10_pH7.4_QMMM_kresoxim-methyl_HB272_babel.pdb

awk '$4=="UNK"' COMPLEX_gold_soln_kresoxim-methyl_m1_3_HBGlu272.pdb > UNK.pdb

pdb4amber -i UNK.pdb -o UNK_clean_1_H.pdb

antechamber -fi pdb -fo prepi -i UNK_clean_1_H.pdb -o UNK_clean_1_H.prepi -rn UNK -c bcc -pf y

parmchk2 -f prepi -i UNK_clean_1_H.prepi -o UNK_1.frcmod

cat 0_80_10_pH7.4_QMMM_kresoxim-methyl_HB272_babel.pdb UNK_clean_1_H.pdb > complex_1.pdb

pdb4amber -i complex_1.pdb -o complex_clean_1.pdb

And now: create 'complex_1_tleap.in' file:

******************** this is 'complex_1_tleap.in' file *************

source leaprc.protein.ff14SB #Source leaprc file for ff14SB protein force field

source leaprc.gaff #Source leaprc file for gaff

source leaprc.water.tip3p #Source leaprc file for TIP3P water model

loadamberprep UNK_clean_1_H.prepi #Load the prepi file for the ligand

loadamberparams UNK_1.frcmod #Load the additional frcmod file for ligand

mol = loadpdb complex_clean_1.pdb #Load PDB file for protein-ligand complex

solvatebox mol TIP3PBOX 10 #Solvate the complex with a cubic water box

addions mol Cl- 6 #Add Cl- ions to neutralize the system

saveamberparm mol complex_clean_1.prmtop complex_clean_1.inpcrd #Save AMBER topology and coordinate files

quit #Quit tleap program

************************ this is 'complex_1_tleap.in' file *************

tleap -s -f complex_1_tleap.in > complex_1_tleap.out

And you get 

complex_clean_1.prmtop

complex_clean_1.inpcrd

And only this one more:

ante-MMPBSA.py -p complex_clean_1.prmtop -c com.prmtop -r rec.prmtop -l LIG.prmtop -s :WAT:Cl- -n :UNK 

You get:

com.prmtop

rec.prmtop

LIG.prmtop

Everything is ready for simulations!!!
