# Supplementary material for: Search for Novel Lead Inhibitors of Yeast Cytochrome bc1, from Drugbank and COCONUT
Source: Molecules. 2021 Jul 16;26(14):4323. doi: 10.3390/molecules26144323 (PMC8307329; doi:10.3390/molecules26144323)
Supplement: Supplementary file 84 [file molecules-26-04323-s001.zip › molecules-1275410 pdf files/Supplementary_files/Supplementary_MD_instructions/Gromacs_input_folder/program_FOR_DETERMINING_the_number_of_H_bonds_AND_hydrophobic_interactions_protein_to_ligand.odt]

R

Ligerr<-read.table("turolig2.csv")

Lig<-data.matrix(Ligerr[,1:3])

tel<-nrow(Lig)/10001

cNO<-c()

cNOr<-c()

cH<-c()

cHr<-c()

for (i in 1:tel) {

if (Ligerr[i,4]=="N" | Ligerr[i,4]=="O") {

cNO<-cbind(cNO,i)

cNOr<-rbind(cNOr,Lig[i,])}

if (Ligerr[i,4]=="H") {

cH<-cbind(cH,i)

cHr<-rbind(cHr,Lig[i,]) }

}

for (k in 1:1) {

if (length(cNO)==1)  {

dist<-array(length(cH))

cbrN<-c()

brH<-0

cbrH<-c()

for (j in 1:length(cH)) {

dist[j]<-sqrt(   ((cNOr[1]-cHr[j,1])^2)+((cNOr[2]-cHr[j,2])^2)+((cNOr[3]-cHr[j,3])^2)  )

if (dist[j]<1.2) {

brH<-brH+1

cbrH<-cbind(cbrH,c(cH[j]))

cbrN<-c(cNO[1])

}

}

}

else {

dist<-matrix(rep(0,length(cH)*length(cNO)),nrow=length(cNO))

cbrN<-c()

brH<-0

cbrH<-c()

for (i in 1:length(cNO)) {

for (j in 1:length(cH)) {

dist[i,j]<-sqrt(   ((cNOr[i,1]-cHr[j,1])^2)+((cNOr[i,2]-cHr[j,2])^2)+((cNOr[i,3]-cHr[j,3])^2)  )

if (dist[i,j]<1.2) {

brH<-brH+1

cbrH<-cbind(cbrH,c(cH[j]))

cbrN<-cbind(cbrN,c(cNO[i]))

}

}

}

}

}

cbrH

cbrN

tel

q()

n

sed -n '2~30p' turolig2.csv >XH1.csv

sed -n '11~24p' turolig2.csv >H1.csv #first number '11' denotes cbrH number from R and each must be called, the second number is tel from R script which is constant.

sed -n '12~24p' turolig2.csv >H2.csv

sed -n '13~24p' turolig2.csv >H3.csv

sed -n '23~24p' turolig2.csv >H4.csv

sed -n '24~24p' turolig2.csv >H5.csv

sed -n '1113~1567p' turoprot.csv >Glu272_N.csv

sed -n '1114~1567p' turoprot.csv >Glu272_H.csv

R

#### SVE OSTALO u R-u !!!!!!!!!!!!!!!!!!!!!!!!!

Ligerr<-read.table("turolig.csv")

Proterr<-read.table("turoprot.csv")

#XH1<-read.table("XH1.csv")

H1<-read.table("H1.csv")

H2<-read.table("H2.csv")

H3<-read.table("H3.csv")

#H4<-read.table("H4.csv")

#H5<-read.table("H5.csv")

#Xh1<-data.matrix(XH1[,1:3])

Hh1<-data.matrix(H1[,1:3]) #crveni dijelovi za ligande s H donorima

Hh2<-data.matrix(H2[,1:3]) #crveni dijelovi za ligande s H donorima

Hh3<-data.matrix(H3[,1:3]) #crveni dijelovi za ligande s H donorima

#Hh4<-data.matrix(H4[,1:3]) #crveni dijelovi za ligande s H donorima

#Hh5<-data.matrix(H5[,1:3]) #crveni dijelovi za ligande s H donorima

cbrN1<-3 # each number is cbrN and must be called IF ligand contains proton donor groups

cbrN2<-6

cbrN3<-7

Lig<-data.matrix(Ligerr[,1:3])

Prot<-data.matrix(Proterr[,1:3])

Protvar<-nrow(Prot)/10001

Ligvar<-nrow(Lig)/10001

dist<-array(rep(NA,100*Protvar*Ligvar),dim=c(100,Protvar,Ligvar))

for (k in 1:100) {

for (i in 1:Protvar) {

for (j in 1:Ligvar) {

dist[k,i,j]<- sqrt(   ((Lig[((k-1)*Ligvar*100)+j,1]-Prot[((k-1)*Protvar*100)+i,1])^2)+((Lig[((k-1)*Ligvar*100)+j,2]-Prot[((k-1)*Protvar*100)+i,2])^2)+((Lig[((k-1)*Ligvar*100)+j,3]-Prot[((k-1)*Protvar*100)+i,3])^2)  )

}}}

Tazerr<-Proterr

Tazvar<-Protvar

for (i in Protvar:1) {

if (min(dist[,i,])>6) {

Tazerr<-Tazerr[-seq((10000*Tazvar+i),i,-Tazvar),]

Tazvar<-Tazvar-1}

}

Taz<-data.matrix(Tazerr[,1:3])

brojac<-0

vec<-c()

dist<-array(rep(NA,10001*Tazvar*Ligvar),dim=c(10001,Tazvar,Ligvar))

for (k in 1:10001) {

for (i in 1:Tazvar) {

for (j in 1:Ligvar) {

dist[k,i,j]<- sqrt(   ((Lig[((k-1)*Ligvar)+j,1]-Taz[((k-1)*Tazvar)+i,1])^2)+((Lig[((k-1)*Ligvar)+j,2]-Taz[((k-1)*Tazvar)+i,2])^2)+((Lig[((k-1)*Ligvar)+j,3]-Taz[((k-1)*Tazvar)+i,3])^2)  )

if ((dist[k,i,j]<3.2)&(Tazerr[i,6]=="N" | Tazerr[i,6]=="O")&(Ligerr[j,4]=="N" | Ligerr[j,4]=="O" | Ligerr[j,4]=="F" | Ligerr[j,4]=="Cl")) {

for (m in 1:3) {

if ((sqrt(   ((Taz[((k-1)*Tazvar)+i,1]-Taz[((k-1)*Tazvar)+i+m,1])^2)+((Taz[((k-1)*Tazvar)+i,2]-Taz[((k-1)*Tazvar)+i+m,2])^2)+((Taz[((k-1)*Tazvar)+i,3]-Taz[((k-1)*Tazvar)+i+m,3])^2)  )<1.2)&&(Tazerr[i+m,6]=="H")&&(sqrt(   ((Lig[((k-1)*Ligvar)+j,1]-Taz[((k-1)*Tazvar)+i+m,1])^2)+((Lig[((k-1)*Ligvar)+j,2]-Taz[((k-1)*Tazvar)+i+m,2])^2)+((Lig[((k-1)*Ligvar)+j,3]-Taz[((k-1)*Tazvar)+i+m,3])^2)  )<2.2)) {

brojac<-brojac+1

vec<-rbind(vec,cbind(k,i,i+m,j))

}

}

if ( (j==cbrN1 | j==cbrN2)&&((sqrt(   ((Taz[((k-1)*Tazvar)+i,1]-Hh1[k,1])^2)+((Taz[((k-1)*Tazvar)+i,2]-Hh1[k,2])^2)+((Taz[((k-1)*Tazvar)+i,3]-Hh1[k,3])^2)  )<2.2) || ( sqrt(   ((Taz[((k-1)*Tazvar)+i,1]-Hh2[k,1])^2)+((Taz[((k-1)*Tazvar)+i,2]-Hh2[k,2])^2)+((Taz[((k-1)*Tazvar)+i,3]-Hh2[k,3])^2)  )<2.2 ) || ( sqrt(   ((Taz[((k-1)*Tazvar)+i,1]-Hh3[k,1])^2)+((Taz[((k-1)*Tazvar)+i,2]-Hh3[k,2])^2)+((Taz[((k-1)*Tazvar)+i,3]-Hh3[k,3])^2)  )<2.2 ) || ( sqrt(   ((Taz[((k-1)*Tazvar)+i,1]-Hh4[k,1])^2)+((Taz[((k-1)*Tazvar)+i,2]-Hh4[k,2])^2)+((Taz[((k-1)*Tazvar)+i,3]-Hh4[k,3])^2)  )<2.2 ) || ( sqrt(   ((Taz[((k-1)*Tazvar)+i,1]-Hh5[k,1])^2)+((Taz[((k-1)*Tazvar)+i,2]-Hh5[k,2])^2)+((Taz[((k-1)*Tazvar)+i,3]-Hh5[k,3])^2)  )<2.2 )) ) {

brojac<-brojac+1

vec<-rbind(vec,cbind(k,i,k,j))} #crveni dijelovi za ligande s H donorima

}

}

}

}

brojac

vec

write.csv(vec,"DB08557.txt")

q()

n

HELP: DEPENDING ON THE PROTON DONOR GROUPS. IF LIGAND HAS NO PROTON DONOR GROUPS THEN THERE IS NO YELLOW HIGHLIGHTED PART OFTTHE CODE ABOVE!

|| ( sqrt(   ((Taz[((k-1)*Tazvar)+i,1]-Hh4[k,1])^2)+((Taz[((k-1)*Tazvar)+i,2]-Hh4[k,2])^2)+((Taz[((k-1)*Tazvar)+i,3]-Hh4[k,3])^2)  )<2.2 )

Some functions to extract specific protein atoms:

sed -n '1115~1567p' turoprot.csv >Glu272_OE1.csv # these numbers must be carefully checked

sed -n '1116~1567p' turoprot.csv >Glu272_OE2.csv

sed -n '1113~1567p' turoprot.csv >Glu272_N.csv

sed -n '1114~1567p' turoprot.csv >Glu272_H.csv

sed -n '565~1567p' turoprot.csv >Tyr132_O_sidechain.csv

R

#### EVERYTHING ELSE IN PROGRAM R !!!!!!!!!!!!!!!!!!!!!!!!!

Ligerr<-read.table("turolig.csv")

Lig<-data.matrix(Ligerr[,1:3])

GluH<-read.table("Glu272_H.csv")

GluN<-read.table("Glu272_N.csv")

H<-data.matrix(GluH[,1:3])

N<-data.matrix(GluN[,1:3])

tel<-nrow(Lig)/nrow(H)

dist<-matrix(rep(NA,nrow(Lig)*2),nrow=nrow(Lig))

brojac<-0

for (i in 1:nrow(H)) {

for (j in 1:tel) {

b<-0

dist[((i-1)*tel)+j,1]<-sqrt( ((Lig[((i-1)*tel)+j,1]-H[i,1])^2)+((Lig[((i-1)*tel)+j,2]-H[i,2])^2)+((Lig[((i-1)*tel)+j,3]-H[i,3])^2) )

if (dist[((i-1)*tel)+j,1]<2.2) {

b<-1 }

dist[((i-1)*tel)+j,2]<-sqrt( ((Lig[((i-1)*tel)+j,1]-N[i,1])^2)+((Lig[((i-1)*tel)+j,2]-N[i,2])^2)+((Lig[((i-1)*tel)+j,3]-N[i,3])^2) )

if ((dist[((i-1)*tel)+j,2]<3.2)&&(b==1)) {

brojac<-brojac+1

b<-0}

}

}

brojac

Ligerr<-read.table("turolig.csv")

Lig<-data.matrix(Ligerr[,1:3])

GluH<-read.table("Glu272_H.csv")

GluN<-read.table("Glu272_N.csv")

H<-data.matrix(GluH[,1:3])

N<-data.matrix(GluN[,1:3])

tel<-nrow(Lig)/nrow(H)

dist<-matrix(rep(NA,nrow(Lig)*2),nrow=nrow(Lig))

brojac<-0

for (i in 1:nrow(H)) {

for (j in 1:tel) {

b<-0

dist[((i-1)*tel)+j,1]<-sqrt( ((Lig[((i-1)*tel)+j,1]-H[i,1])^2)+((Lig[((i-1)*tel)+j,2]-H[i,2])^2)+((Lig[((i-1)*tel)+j,3]-H[i,3])^2) )

if (dist[((i-1)*tel)+j,1]<2.6) {

b<-1 }

dist[((i-1)*tel)+j,2]<-sqrt( ((Lig[((i-1)*tel)+j,1]-N[i,1])^2)+((Lig[((i-1)*tel)+j,2]-N[i,2])^2)+((Lig[((i-1)*tel)+j,3]-N[i,3])^2) )

if ((dist[((i-1)*tel)+j,2]<3.6)&&(b==1)) {

brojac<-brojac+1

b<-0}

}

}

brojac

Ligerr<-read.table("turolig.csv")

Lig<-data.matrix(Ligerr[,1:3])

GluH<-read.table("Glu272_H.csv")

GluN<-read.table("Glu272_N.csv")

H<-data.matrix(GluH[,1:3])

N<-data.matrix(GluN[,1:3])

tel<-nrow(Lig)/nrow(H)

dist<-matrix(rep(NA,nrow(Lig)*2),nrow=nrow(Lig))

brojac<-0

vec<-c()

for (i in 1:nrow(H)) {

for (j in 1:tel) {

b<-0

dist[((i-1)*tel)+j,1]<-sqrt( ((Lig[((i-1)*tel)+j,1]-H[i,1])^2)+((Lig[((i-1)*tel)+j,2]-H[i,2])^2)+((Lig[((i-1)*tel)+j,3]-H[i,3])^2) )

if (dist[((i-1)*tel)+j,1]<3.0) {

b<-1 }

dist[((i-1)*tel)+j,2]<-sqrt( ((Lig[((i-1)*tel)+j,1]-N[i,1])^2)+((Lig[((i-1)*tel)+j,2]-N[i,2])^2)+((Lig[((i-1)*tel)+j,3]-N[i,3])^2) )

if ((dist[((i-1)*tel)+j,2]<4.0)&&(b==1)) {

brojac<-brojac+1

vec<-cbind(vec,c(i))

b<-0}

}

}

brojac

Ligerr<-read.table("turolig2.csv")

Lig<-data.matrix(Ligerr[,1:3])

Tyr132Os<-read.table("Tyr132_O_sidechain.csv")

O<-data.matrix(Tyr132Os[,1:3])

tel<-nrow(Lig)/nrow(O)

dist<-array(nrow(Lig))

brojac<-0

for (i in 1:nrow(O)) {

for (j in 1:tel) {

dist[((i-1)*tel)+j]<-sqrt( ((Lig[((i-1)*tel)+j,1]-O[i,1])^2)+((Lig[((i-1)*tel)+j,2]-O[i,2])^2)+((Lig[((i-1)*tel)+j,3]-O[i,3])^2) )

if (dist[((i-1)*tel)+j]<3.5) {

brojac<-brojac+1

b<-0}

}

}

brojac

fg="$(grep "L3" L3_tr.pdb | awk '{print $7,$8,$9,$12}')"

echo "$fg" > turolig0.csv

fg="$(grep "CZ  PHE A 129" md_0_10_no_L3.pdb | awk '{print $7,$8,$9}')"

echo "$fg" >  turoCZ_PHE129.csv

fg="$(grep "CG  PHE A 129" md_0_10_no_L3.pdb | awk '{print $7,$8,$9}')"

echo "$fg" >  turoCG_PHE129.csv

fg="$(grep "CD  ILE A 125" md_0_10_no_L3.pdb | awk '{print $7,$8,$9}')"

echo "$fg" >  turoCD_ILE125.csv

fg="$(grep "CA  GLY A 143" md_0_10_no_L3.pdb | awk '{print $7,$8,$9}')"

echo "$fg" >  turoCA_GLY143.csv

fg="$(grep "CZ  PHE A 278" md_0_10_no_L3.pdb | awk '{print $7,$8,$9}')"

echo "$fg" >  turoCZ_PHE278.csv

fg="$(grep "CG  PHE A 278" md_0_10_no_L3.pdb | awk '{print $7,$8,$9}')"

echo "$fg" >  turoCG_PHE278.csv

fg="$(grep "CE  MET A 295" md_0_10_no_L3.pdb | awk '{print $7,$8,$9}')"

echo "$fg" >  turoCE_MET295.csv

mkdir TUROC

mv turoC* TUROC

mv turolig0.csv TUROC

mv TUROC/* .

R

#### EVERYTHING ELSE IN PROGRAM R !!!!!!!!!!!!!!!!!!!!!!!!!

Ligerr<-read.table("turolig0.csv")

Lig<-data.matrix(Ligerr[,1:3])

Phe129CZ<-read.table("turoCZ_PHE129.csv")

Phe<-data.matrix(Phe129CZ[,1:3])

tel<-nrow(Lig)/nrow(Phe)

dist<-array(nrow(Lig))

brojac129CZ<-0

for (i in 1:nrow(Phe)) {

for (j in 1:tel) {

dist[((i-1)*tel)+j]<-sqrt( ((Lig[((i-1)*tel)+j,1]-Phe[i,1])^2)+((Lig[((i-1)*tel)+j,2]-Phe[i,2])^2)+((Lig[((i-1)*tel)+j,3]-Phe[i,3])^2) )

if (dist[((i-1)*tel)+j]<3.5) {

brojac129CZ<-brojac129CZ+1 }

}

}

brojac129CZ

Phe129CG<-read.table("turoCG_PHE129.csv")

Phe<-data.matrix(Phe129CG[,1:3])

dist<-array(nrow(Lig))

brojac129CG<-0

for (i in 1:nrow(Phe)) {

for (j in 1:tel) {

dist[((i-1)*tel)+j]<-sqrt( ((Lig[((i-1)*tel)+j,1]-Phe[i,1])^2)+((Lig[((i-1)*tel)+j,2]-Phe[i,2])^2)+((Lig[((i-1)*tel)+j,3]-Phe[i,3])^2) )

if (dist[((i-1)*tel)+j]<3.5) {

brojac129CG<-brojac129CG+1 }

}

}

brojac129CG

ILE125<-read.table("turoCD_ILE125.csv")

Phe<-data.matrix(ILE125[,1:3])

dist<-array(nrow(Lig))

brojac125<-0

vec<-c()

for (i in 1:nrow(Phe)) {

for (j in 1:tel) {

dist[((i-1)*tel)+j]<-sqrt( ((Lig[((i-1)*tel)+j,1]-Phe[i,1])^2)+((Lig[((i-1)*tel)+j,2]-Phe[i,2])^2)+((Lig[((i-1)*tel)+j,3]-Phe[i,3])^2) )

if (dist[((i-1)*tel)+j]<3.5) {

brojac125<-brojac125+1

vec<-cbind(vec,c(i))}

}

}

brojac125

GLY143<-read.table("turoCA_GLY143.csv")

Phe<-data.matrix(GLY143[,1:3])

dist<-array(nrow(Lig))

tel<-nrow(Lig)/nrow(Phe)

brojac143<-0

vec<-c()

for (i in 1:nrow(Phe)) {

for (j in 1:tel) {

dist[((i-1)*tel)+j]<-sqrt( ((Lig[((i-1)*tel)+j,1]-Phe[i,1])^2)+((Lig[((i-1)*tel)+j,2]-Phe[i,2])^2)+((Lig[((i-1)*tel)+j,3]-Phe[i,3])^2) )

if (dist[((i-1)*tel)+j]<3.5) {

brojac143<-brojac143+1

vec<-cbind(vec,c(i))}

}

}

brojac143

PHE278CZ<-read.table("turoCZ_PHE278.csv")

Phe<-data.matrix(PHE278CZ[,1:3])

dist<-array(nrow(Lig))

brojac278CZ<-0

for (i in 1:nrow(Phe)) {

for (j in 1:tel) {

dist[((i-1)*tel)+j]<-sqrt( ((Lig[((i-1)*tel)+j,1]-Phe[i,1])^2)+((Lig[((i-1)*tel)+j,2]-Phe[i,2])^2)+((Lig[((i-1)*tel)+j,3]-Phe[i,3])^2) )

if (dist[((i-1)*tel)+j]<3.5) {

brojac278CZ<-brojac278CZ+1 }

}

}

brojac278CZ

PHE278CG<-read.table("turoCG_PHE278.csv")

Phe<-data.matrix(PHE278CG[,1:3])

dist<-array(nrow(Lig))

brojac278CG<-0

for (i in 1:nrow(Phe)) {

for (j in 1:tel) {

dist[((i-1)*tel)+j]<-sqrt( ((Lig[((i-1)*tel)+j,1]-Phe[i,1])^2)+((Lig[((i-1)*tel)+j,2]-Phe[i,2])^2)+((Lig[((i-1)*tel)+j,3]-Phe[i,3])^2) )

if (dist[((i-1)*tel)+j]<3.5) {

brojac278CG<-brojac278CG+1 }

}

}

brojac278CG

MET295<-read.table("turoCE_MET295.csv")

Phe<-data.matrix(MET295[,1:3])

dist<-array(nrow(Lig))

brojac295<-0

for (i in 1:nrow(Phe)) {

for (j in 1:tel) {

dist[((i-1)*tel)+j]<-sqrt( ((Lig[((i-1)*tel)+j,1]-Phe[i,1])^2)+((Lig[((i-1)*tel)+j,2]-Phe[i,2])^2)+((Lig[((i-1)*tel)+j,3]-Phe[i,3])^2) )

if (dist[((i-1)*tel)+j]<3.5) {

brojac295<-brojac295+1 }

}

}

brojac295

brojac129CZ

brojac129CG

brojac125

brojac143

brojac278CZ

brojac278CG

brojac295
